# Practical Recommendations for Optimal Thromboprophylaxis in Patients with COVID-19: A Consensus Statement Based on Available Clinical Trials

**DOI:** 10.3390/jcm11205997

**Published:** 2022-10-11

**Authors:** Konstantinos G. Kyriakoulis, Evangelos Dimakakos, Ioannis G. Kyriakoulis, Mariella Catalano, Alex C. Spyropoulos, Sam Schulman, James Douketis, Anna Falanga, Anthony Maraveyas, Dan-Mircea Olinic, Jill Belch, Grigorios Gerotziafas, Konstantinos Syrigos, Anastasios Kollias, COVID-19 Thrombosis Collaborative Group, Endorsed by VAS-European Independent Foundation in Angiology/Vascular Medicine, UEMS Division of Angiology/Vascular Medicine/and ESVM-European Society of Vascular Medicine and Supported by the Balkan Working Group for Prevention and Treatment of Venous Thromboembolism

**Affiliations:** 1 Third Department of Medicine, School of Medicine, Sotiria Hospital, National and Kapodistrian University of Athens, 11527 Athens, Greece; 2 Inter-University Research Center on Vascular Disease, Department of Biomedical Science, L Sacco Hospital, University of Milan, 20157 Milan, Italy; 3 Feinstein Institutes for Medical Research, The Donald and Barbara Zucker School of Medicine at Hofstra/Northwell, Hempstead, NY 11549, USA; 4 Department of Medicine Northwell Health, Lenox Hill Hospital, New York, NY 10075, USA; 5 Department of Medicine, Thrombosis and Atherosclerosis Research Institute, McMaster University, Hamilton, ON L8S 4L8, Canada; 6 Department of Medicine, St. Joseph’s Healthcare Hamilton, McMaster University, Hamilton, ON L8S 4L8, Canada; 7 School of Medicine, University of Milan Bicocca, 20126 Milan, Italy; 8 Department of Immunohematology and Transfusion Medicine, Hospital Papa Giovanni XXIII, 24127 Bergamo, Italy; 9 Queens Centre Oncology & Hematology, Faculty of Health Sciences, Hull York Medical School, Cottingham, Hull HU6 7RU, UK; 10 Medical Clinic No. 1, “Iuliu Hatieganu” University of Medicine and Pharmacy, 400347 Cluj-Napoca, Romania; 11 Institute of Cardiovascular Research, Ninewells Hospital and Medical School, University of Dundee, Dundee DD1 9SY, UK; 12 Sorbonne University, Institut National de la Santé et de la Recherche Médicale (INSERM), Unit 938, Research Group Cancer, Biology and Therapeutics, Centre de Recherche Saint-Antoine (CRSA), Institut Universitaire de Cancérologie, 75012 Paris, France; 13 Thrombosis Center, Tenon-Saint Antoine, Hôpitaux Universitaires de l’Est Parisien, Assistance Publique Hôpitaux de Paris (APHP), 75012 Paris, France

**Keywords:** anticoagulation, COVID-19, COVID-19 therapeutics, dosage, mortality, thromboprophylaxis, treatment

## Abstract

Coronavirus disease 2019 (COVID-19) has been shown to be strongly associated with increased risk for venous thromboembolism events (VTE) mainly in the inpatient but also in the outpatient setting. Pharmacologic thromboprophylaxis has been shown to offer significant benefits in terms of reducing not only VTE events but also mortality, especially in acutely ill patients with COVID-19. Although the main source of evidence is derived from observational studies with several limitations, thromboprophylaxis is currently recommended for all hospitalized patients with acceptable bleeding risk by all national and international guidelines. Recently, high quality data from randomized controlled trials (RCTs) further support the role of thromboprophylaxis and provide insights into the optimal thromboprophylaxis strategy. The aim of this statement is to systematically review all the available evidence derived from RCTs regarding thromboprophylaxis strategies in patients with COVID-19 in different settings (either inpatient or outpatient) and provide evidence-based guidance to practical questions in everyday clinical practice. Clinical questions accompanied by practical recommendations are provided based on data derived from 20 RCTs that were identified and included in the present study. Overall, the main conclusions are: (i) thromboprophylaxis should be administered in all hospitalized patients with COVID-19, (ii) an optimal dose of inpatient thromboprophylaxis is dependent upon the severity of COVID-19, (iii) thromboprophylaxis should be administered on an individualized basis in post-discharge patients with COVID-19 with high thrombotic risk, and (iv) thromboprophylaxis should not be routinely administered in outpatients. Changes regarding the dominant SARS-CoV-2 variants, the wide immunization status (increasing rates of vaccination and reinfections), and the availability of antiviral therapies and monoclonal antibodies might affect the characteristics of patients with COVID-19; thus, future studies will inform us about the thrombotic risk and the optimal therapeutic strategies for these patients.

## 1. Introduction

The relationship between the Coronavirus disease 2019 (COVID-19) and venous thromboembolism (VTE) was first reported as a case report in March 2020, close to the onset of the pandemic [1]. Since then, an enormous amount of evidence has emerged and nearly ten thousand articles on COVID-19 and VTE have been published within the last two years [2]. COVID-19 is associated with an increased VTE risk [3] that can be attributed to factors related to (i) the virus and the induced thromboinflammation observed in severe infection per se; (ii) the hospitalization conditions (immobilization); and (iii) the individual patient risk factors for VTE, most of which are also risk factors for severe COVID-19 [4].

While the pathophysiological mechanisms are not clearly defined, hospitalized patients with severe COVID-19 exhibit an increased inflammatory status both at the systemic (cytokine storm) and local (endothelial injury with thromboinflammation) level [5,6,7]. COVID-19 associated coagulopathy mainly manifests with a prothrombotic tendency, as platelet count is preserved, coagulation function tests are normal or minimally prolonged, and bleeding events are uncommon [8]. These features can be distinguished from a diagnosis of disseminated intravascular coagulation (DIC), which can occur in patients with critical infectious illness [8]. Interestingly, COVID-19 associated coagulopathy and the related microthrombi formation mainly affects the lung vessels, as confirmed by autopsy studies [5,9].

The prevalence of pulmonary embolism (PE) and deep vein thrombosis (DVT) in hospitalized patients with COVID-19 varies widely and is likely due to across study differences in patient characteristics and VTE diagnostic and screening protocols [4]. In a meta-analysis of 47 studies (n = 6459 patients), where all patients were subjected to imaging diagnostic evaluation for PE/DVT, the prevalence of PE and DVT in hospitalized patients with COVID-19 was about 32% and 27%, respectively [10]. Importantly, a two-fold increased risk for death was demonstrated in patients with VTE compared to those without VTE [10].

Considering the increased VTE risk of COVID-19 and the association between VTE and mortality, it is not surprising that pharmacologic thromboprophylaxis has been shown to offer significant benefits in terms of reducing not only VTE events but also mortality, especially in cases of severe COVID-19 [11,12,13,14]. Thus, thromboprophylaxis is currently recommended by multiple national and international clinical practice guidelines for hospitalized patients with an acceptable bleeding risk [15,16,17,18,19,20,21]. Yet, the main source of evidence has been derived from observational studies with important methodological limitations. Recently, randomized trials have investigated the role of thromboprophylaxis and provide insights into the optimal thromboprophylaxis strategy.

The aim of this statement is to systematically review all the available evidence derived from randomized clinical trials (RCTs) regarding the role of thromboprophylaxis in adult patients with COVID-19 (both in the inpatient and outpatient setting), to address specific key questions, and to transform this evidence into practical lessons to be implemented in daily clinical practice.

## 2. Materials and Methods

A systematic PubMed search was conducted in line with the Preferred Reporting Items for Systematic Reviews and Meta-Analyses (PRISMA) recommendations independently by two investigators (KGK and IGK) [22]. The literature search was conducted using the algorithm (“coronavirus 2019” OR “2019-nCoV” OR “SARS-CoV-2” OR “COVID-19” OR COVID OR COVID19) AND (thrombotic OR thrombosis OR “deep vein” OR “pulmonary embolism” OR thromboemboli* OR heparin) AND randomi* until August 10, 2022. Articles were also identified from references of relevant articles using the snowball procedure. Disagreements were resolved by consensus with senior authors. Eligible studies were RCTs regarding different thromboprophylaxis strategies in patients with COVID-19 in different settings (either inpatient or outpatient). Data concerning the population characteristics, the interventions/comparators, and the main conclusions of each RCT were extracted and tabulated.

## 3. Results—Key Questions and Practical Recommendations

Among the 352 articles initially retrieved, 20 fulfilled the inclusion criteria and were included in the systematic review (Table 1). Clinical questions accompanied by practical recommendations were formed according to available data derived from the included RCTs.

### 3.1. Hospitalized Patients

#### 3.1.1. Does Thromboprophylaxis Offer a Benefit to All Hospitalized Patients with COVID-19?

Medically ill patients with infectious diseases requiring hospitalization usually receive thromboprophylaxis based upon their VTE and bleeding risk [23]. Given the increased risk for VTE in hospitalized patients with COVID-19, thromboprophylaxis seems a reasonable approach; yet no RCT comparing thromboprophylaxis versus placebo was identified. Despite this lack, the evidence from large-scale observational studies is consistent and in favor of thromboprophylaxis in hospitalized patients, and this has been translated into recommended practice [16,19].

In fact, the earliest evidence is derived from an observational study that reported decreased mortality in patients with COVID-19, who received thromboprophylaxis with low-molecular-weight heparin (LMWH), compared with those who did not [14]. Additional studies reporting beneficial effects of anticoagulant prophylaxis in patients with COVID-19 have subsequently been published [11,12,13]. A cohort study of 4297 hospitalized patients with COVID-19 showed that the early (within 24 h of hospitalization) initiation of thromboprophylaxis versus no anticoagulation resulted in a 27% decreased risk for 30-day mortality for those receiving anticoagulation [12]. In this study, 70% of patients received LMWH [12]. In another study, no anticoagulation was associated with increased risk for the composite outcome of death, VTE, intensive care unit (ICU) admission compared with LMWH use, irrespective of the dose intensity (prophylactic, intermediate, or therapeutic dosages) [11]. In the latter study, thromboprophylaxis use was additionally associated with a significant decrease in acute phase inflammatory indices such as ferritin, interferon gamma, or interleukin-6 [11].


***Conclusion—Recommendation:***
*Thromboprophylaxis is associated with survival benefit (low dose compared to no thromboprophylaxis) and is recommended for all hospitalized patients with COVID-19 with an acceptable bleeding risk profile.*


#### 3.1.2. Which Is the Drug of Choice for Inpatient Thromboprophylaxis?

Among all the anticoagulants, LMWH is the most studied drug that has been used for thromboprophylaxis in hospitalized patients with COVID-19 and is currently recommended as the first option by most guidance reports [16]. Unfractionated heparin (UFH) and fondaparinux are considered when LMWH is contraindicated (e.g., UFH in severe renal failure or fondaparinux in patients with history of heparin-induced thrombocytopenia, respectively) [16]. The majority of RCTs examining thromboprophylaxis strategies (16 out of 20) reported in Table 1 included interventions mainly with LMWH, especially enoxaparin. Head-to-head comparison of LMWH with direct oral anticoagulants (DOACs) has not been done but indirect data can be extracted from the AntiCoagulaTIon cOroNa virus (ACTION) trial [24]. In this randomized study, therapeutic versus prophylactic dosage of thromboprophylaxis was compared among 615 hospitalized patients with COVID-19 [24]. A total of 90% of the therapeutic arm received rivaroxaban, while 84% of the prophylactic arm received LMWH. No statistically significant difference was observed in the primary efficacy outcome (any VTE, myocardial infarction, stroke, systemic embolism, and major adverse limb events) but bleeding events were more frequent in the therapeutic rivaroxaban arm [24]. Conclusions regarding the comparison of LMWH and DOACs cannot be drawn since different dosages were implemented and different durations of treatment were planned (i.e., inpatient administration of prophylactic dose LMWH but up to 30-days post-discharge for therapeutic dose rivaroxaban) [24].

LMWH is the established drug class of choice in hospitalized patients with COVID-19 because of its anticoagulant effects coupled with putative pleiotropic anti-viral and anti-inflammatory properties [25]. LMWH has an important role in suspending the entry of the virus into the host cells and in modulating the inflammatory state and cytokine storm [11,25]. Moreover, it seems to present the least interactions with anti-viral or other drugs used in the treatment of COVID-19 infection [26,27,28] compared to other anticoagulants. Importantly, for hospitalized patients that are already treated with oral anticoagulants (vitamin K antagonists [VKA] or DOACs), a switch to LMWH can be considered (and is preferred in critical disease) because of the fewer potential drug–drug and drug–food interactions [26,27,28]. A recent meta-analysis showed that the prevalence of new-onset atrial fibrillation in hospitalized patients with COVID-19 was 7.4% [29]. LMWH can be suggested as the preferred anticoagulation regimen for hospitalized patients with COVID-19 and new-onset atrial fibrillation with a high CHA₂DS₂-VASc score, especially those with critical disease, mainly due to the abovementioned fewer interactions, whereas DOACs would be preferred for long-term anticoagulation afterwards [30]. A recent Good Practice Guidance Statement by the International Society on Thrombosis and Haemostasis (ISTH) also recommends LMWH as the anticoagulant of choice for hospitalized patients with COVID-19 [31].


***Conclusion—Recommendation:***
*LMWH has the largest body of evidence regarding the beneficial role of thromboprophylaxis in hospitalized patients with COVID-19 and should be currently regarded as the drug of choice.*


#### 3.1.3. What Is the Optimal Dosage for Inpatient Thromboprophylaxis? What Is the Role of Timing of Thromboprophylaxis Initiation?

Anticoagulation options include prophylactic dose, intermediate dose (doses higher than the prophylactic ones but lower than the therapeutic ones), and therapeutic dose anticoagulant regimens. Initial guidance recommendations relating to COVID-19 favored prophylactic dose regimens with higher doses being considered for selected patients, such as those with severe disease [16].

Several RCTs have addressed the issue of the optimal anticoagulant dosage for hospitalized patients with COVID-19 (Table 2). The Intermediate vs. Standard-Dose Prophylactic Anticoagulation in Critically ill Patients With COVID-19: An Open Label Randomized Controlled Trial (INSPIRATION) was the first RCT that addressed this issue comparing intermediate versus prophylactic dosages in patients with COVID-19 admitted to the ICU [32]. The findings of this trial did not show any benefit for the intermediate over standard prophylactic dosage either in the primary analysis [32] or in the 90-day follow-up sub-analysis [33]. In their conclusion, the authors recommended against the routine empirical use of intermediate dosage anticoagulation in patients with COVID-19 admitted to the ICU. However, it is important to mention that this was an open-label trial and patients were randomized 12 days (median) after the onset of symptoms with details regarding their previous anticoagulation regimens missing. This fine point is of potential importance since recent data support the idea that timing of initiation of anticoagulation may be equally important as optimal dosage and therefore the results should be interpreted with caution [34].

In line with the above assumption, the multiplatform RCT combining Randomized, Embedded, Multifactorial Adaptive Platform Trial for Community-Acquired Pneumonia (REMAP-CAP), A Multicenter, Adaptive, Randomized Controlled Platform Trial of the Safety and Efficacy of Antithrombotic Strategies in Hospitalized Adults with COVID-19 (ACTIV-4a) and Antithrombotic Therapy to Ameliorate Complications of COVID-19 (ATTACC) (REMAP-CAP, AC-TIV-4a, and ATTACC), showed a benefit of the therapeutic versus prophylactic dosage only when the former was administered to non-critically ill patients [35]. The same study group failed to prove a similar benefit when the comparison was made in the setting of critically ill patients [36]. The importance of the prompt initiation of the increased dosages in high-risk patients has been implied, to gain benefit from this intervention [35,36]. The HEP-COVID trial demonstrated a reduction in the composite endpoint of major thromboembolic events and mortality in selected non-ICU patients with highly elevated (>4 × ULN) D-dimer levels or a sepsis-induced coagulopathy (SIC) score ≥4 receiving therapeutic versus lower dosages [37]. Once more, the beneficial effect of the therapeutic dosage was not demonstrated in ICU patients. The Therapeutic Anticoagulation versus Standard Care as a Rapid Response to the COVID-19 Pandemic (RAPID) trial showed similar results in reduction of the secondary outcome of all-cause mortality at 28 days in moderately ill patients with increased D-dimer levels [38]. Moreover, the small HESACOVID trial revealed a decreased need for mechanical ventilation and improved gas exchange in patients with severe COVID-19 receiving therapeutic enoxaparin compared to standard prophylactic anticoagulation [39]. In the same context, Oliynuk et al. conducted a small, randomized trial comparing prophylactic enoxaparin versus therapeutic enoxaparin or UFH [40]. Hospitalized ICU patients that were not intubated prior to study enrollment were included. The authors concluded that there was an increased risk for intubation or death in the prophylactic enoxaparin treatment arm compared to the therapeutic dosage treatment groups. On the other hand, the results of the AntiCoagulaTIon cOroNavirus (ACTION) trial do not support the use of therapeutic doses due to no improvement in clinical outcomes and increased bleeding events with therapeutic over prophylactic dosages [24]. It is noteworthy that in the ACTION trial, randomization was done up to 14 days after the onset of symptoms with previous anticoagulation status remaining unclear. Notably, the majority of patients in the therapeutic arm (90%) received rivaroxaban while patients in the prophylactic arm received enoxaparin (85%) or UFH (15%). It is also noteworthy that the therapeutic arm was treated for 30 days after hospital discharge while prophylactic anticoagulation was administered only during the hospital stay. Additionally, COVID-HEP included 159 patients with COVID-19 (28% in ICU setting) and compared therapeutic versus prophylactic dose for acutely ill and intermediate versus prophylactic dose for critically ill patients [41]. Both higher anticoagulation dosages failed to offer clinical benefits; however, the study was prematurely discontinued due to low recruitment rate [41]. The BEMICOP study compared therapeutic versus prophylactic dosage of bemiparin in 65 moderately ill patients with increased D-dimer and failed to demonstrate a protective role of therapeutic dosage [42]. Perepu et al. did not demonstrate a significant benefit of the intermediate dose over prophylactic dose heparin in both ICU and non-ICU patients; however, in 61% of the study sample, obesity and weight-adjusted doses were used (obese patients in the standard dose arm received either 30 mg or 40 mg of enoxaparin twice daily whereas in the intermediate dose arm, all obese patients received 0.5 mg/kg twice daily) [43]. Finally, the small X-COVID trial showed a potential benefit of the intermediate over prophylactic dose heparin but was underpowered and prematurely discontinued [44] (Table 2).

Apart from data derived from RCTs, some observational studies demonstrated a benefit to patients receiving higher than prophylactic dose regimens [11,13,45,46]. Interestingly, a recent meta-analysis reported a trend for fewer VTE events with increasing dosages of anticoagulation [47]. However, observational studies are inevitably subjected to several forms of bias—including indication bias and selection bias from lack of randomization. Thus, patients with more severe disease usually tend to receive more intense therapeutic interventions, the beneficial impact of which may be hard to determine. This indication bias has been shown in a recent meta-analysis, where a trend for survival benefit was observed for the therapeutic over prophylactic dose only in the adjusted (for several confounders) analyses, while the opposite trend was revealed for the unadjusted analysis [46]. Interestingly, in the same meta-analysis a survival benefit was shown for intermediate over prophylactic dose heparin regimens [46].

Most guidelines initially recommended prophylactic dose anticoagulation for hospitalized patients and the consideration of a higher dose regimen in those at increased VTE risk [16]. The most recent formal guidelines using accepted methodology from the ISTH [17] and guidance from American College of Chest Physicians (ACCP/CHEST) [21] provide an updated approach based on recent findings from RCTs and meta-analyses [48]. The CHEST clinical guidance suggests that the severity of COVID-19 should be assessed before a decision for thromboprophylaxis [21]. The ISTH Guidelines propose that for hospitalized non-critically ill patients at increased risk for VTE [e.g., elevated D-dimer levels (>2 × ULN) or with need for oxygen requirements or low baseline oxygenation] with low bleeding risk, therapeutic dosage thromboprophylaxis is recommended. If a therapeutic dosage cannot be administered, a prophylactic (and not intermediate) dosage should be considered [17,21]. On the other hand, in critically ill patients (ICU) or those in a step-down or ward setting receiving high-flow nasal cannula oxygenation, prophylactic over intermediate or therapeutic dose heparin is recommended [20,21]. The National Institute for Health (NIH) [20] and the American Society of Hematology (ASH) guidance documents [15] are aligned with these recommendations. The NIH guidance and ISTH guidelines documents further recommend decreasing the anticoagulation intensity in the case of clinical deterioration when a patient changes from acutely to critically ill [17,20].

The CHEST guidance document discourages the use of intermediate dose anticoagulation based on lack of supportive RCT evidence and the potential for dose regimen confusion in clinical practice. It should be noted that intermediate dosages have been traditionally used in both observational [47] and randomized studies (Table 2). Three RCTs exclusively used intermediate dosages compared with prophylactic anticoagulation dosages [32,43,44]. Two of them were conducted mainly in an ICU setting and did not demonstrate any clinical benefit [32,43]; however, one of these trials was conducted in general wards and showed a marginal benefit in favor of the intermediate dosage [44]. Other studies have used mixed dosage strategies and consequently possible positive effects of intermediate dosages may have been blunted [35] (Table 2). In a recent meta-analysis including both data from RCTs and observational studies, but with the latter providing adjusted analyses for confounders, a beneficial effect of the intermediate over prophylactic dosage was observed, especially in the non-ICU setting [46]. It should be highlighted that the intermediate dosage is understudied in RCTs including acutely ill non-ICU or ward patients. Thus, at present, current data from RCTs support the use of a therapeutic dosage in acutely ill non-ICU or ward patients, discourage an escalation strategy with worsening status, and suggest a prophylactic dosage in critically ill patients, especially in the ICU.
***Conclusion-Recommendation:****All hospitalized patients with COVID-19 should at least receive timely prophylactic anticoagulation. In the case of high risk for bleeding/active bleeding, mechanical prophylaxis should be used.**In high thrombotic risk, non-critically ill (non-ICU) patients, a therapeutic dose of heparin (LMWH/UFH) is recommended, taking into consideration the individual patient’s bleeding risk. The role of the intermediate dose heparin in such patients has not been adequately studied in RCTs.**For critically ill (ICU) patients, higher dosages do not offer a benefit and increase the bleeding risk; therefore, a prophylactic dosage should be administered, preferably with LMWH/UFH.*

#### 3.1.4. What Is the Role of the Antiplatelet Therapy in Hospitalized Patients with COVID-19 in the Context of Thromboprophylaxis? What about Patients Already on Antiplatelet Treatment?

Antiplatelet drugs are not recommended for thromboprophylaxis in general. Four RCTs evaluated the role of antiplatelet drugs in hospitalized COVID-19 patients [49,50,51] and outpatients [52] without demonstrating any significant benefit (Table 1). It should be noted that in these trials, hospitalized patients were already receiving anticoagulation for thromboprophylaxis in various dosages.

Regarding patients already receiving antiplatelet drugs, the following should be taken into consideration: (i) the indication of the antiplatelet treatment (secondary cardiovascular prevention—strong evidence; primary cardiovascular prevention—weak evidence [53]); (ii) the thrombotic and bleeding risk; and (iii) the benefit/safety of the co-administration of complex antiplatelet regimens and anticoagulants (e.g., dual antiplatelet treatment after a recent acute coronary event or percutaneous coronary intervention—in this case additional prophylactic thromboprophylaxis should be considered in addition to antiplatelet regimen, on an individualized basis and with periodic assessment of the bleeding risk). According to the recent Good Practice Guidance Statement by the ISTH, add-on antiplatelet therapy should not be routinely initiated in hospitalized patients with COVID-19 [31]. The exception could be in critically ill patients with COVID-19, with a low risk for bleeding, and treated with prophylactic dose LMWH and gastric protection with a proton pump inhibitor. In this subset, the addition of antiplatelet therapy (aspirin 81 mg or clopidogrel 75 mg daily) might reduce mortality at 90 days after discharge, as shown in the REMAP-CAP trial [50].


***Conclusion—Recommendation:***
*Antiplatelet drugs should not be routinely initiated for thromboprophylaxis and concomitant administration with anticoagulants should be considered on an individualized basis, taking into consideration the indication for antiplatelet treatment and the thrombotic/bleeding risk of each patient.*


#### 3.1.5. What Is the Bleeding Risk Associated with Thromboprophylaxis?

Thromboprophylaxis is widely regarded in most patients as having a net therapeutic benefit when balancing efficacy (to prevent thrombosis) and safety (bleeding risk), whereas mechanical methods of thromboprophylaxis are recommended only in a minority of patients with high bleeding risk [16]. Risk factors for bleeding are patient-specific and include age, underlying disease severity (e.g., COVID-19- or sepsis-associated coagulopathy), comorbidities (e.g., impaired renal or hepatic function), as well as the type and intensity of anticoagulant used.

An important part of the RCTs’ objectives was not only to address the efficacy of thromboprophylaxis interventions, but also to verify the safety of these strategies in terms of clinically significant and important major bleeding events. The majority of the RCTs demonstrated the low bleeding risk of the thromboprophylaxis strategies (Table 1). Two trials in non-ICU patients demonstrated increased major bleeding events with therapeutic dosages [24,35]. Another two trials using antiplatelet drugs in addition to thromboprophylaxis anticoagulation found that this intervention was associated with increased incidence of bleeding events [49,50].


***Conclusion-Recommendation:***
*Thromboprophylaxis should be regarded as a clinically beneficial and low bleeding risk intervention for most hospitalized patients with COVID-19. Detailed individualized bleeding risk assessment should be conducted, especially in cases where increased dosages are considered.*


### 3.2. Outpatients and Post-Discharge Patients—Practical Considerations for Outpatients and Post-Discharge Patients

The question of whether outpatients and post-discharge patients with COVID-19 should receive thromboprophylaxis was raised early. COVID-19 associated coagulopathy was more thoroughly investigated and a proportion of COVID-19 mortality was largely attributed to thrombotic events. Moreover, the main impetus for post-discharge prophylaxis was the premise that the at-risk period persists after hospitalization. Additionally, using anticoagulants in ambulatory patients with COVID-19 could possibly attenuate the pneumonitis and ventilation/perfusion (V/Q) mismatch related to inflammation and microthrombi. Nevertheless, inconclusive data were primarily available and only one third of the available guidance reports referred to outpatients and post-discharge patients, mainly recommending non-pharmacological thromboprophylaxis measures (e.g., increased mobilization and hydration) [16]. During the pandemic, it was demonstrated that thrombotic events tend to occur early in the clinical course of COVID-19 [54]. Moreover, in the outpatient setting, the incidence of VTE is higher among outpatients with certain characteristics (older age, male sex, obesity, inherited thrombophilia, no or partial vaccination) [55]. In this context, early initiation of thromboprophylaxis in outpatients with adverse prognostic factors for severe disease (candidates for hospitalization) and increased VTE risk could be regarded as a reasonable approach. With the increased use of oral antivirals such as Paxlovid (nirmatrelvir/ritonavir) for outpatients at high risk for COVID-19 progression, the co-administration of anticoagulants can be problematic because many DOACs share the same (CYP-450) metabolic pathway as ritonavir (which, in fact, is used to increase the bioavailability of the active anti-coronavirus agent nirmatrelvir), with the potential for DOAC bioaccumulation and an increased bleeding risk. Management options in anticoagulated patients who require Paxlovid include reducing the dose of the DOAC, using a DOAC with less drug-drug interaction potential (e.g., edoxaban), or switching to a LMWH [28]. Five RCTs have addressed the question of outpatient thromboprophylaxis [52,56,57,58,59].

The first randomized trial that assessed the efficacy and safety of an antithrombotic agent in the outpatient setting was the study by Gonzalez-Ochoa et al. [57]. The investigators randomized 243 outpatients at high risk for severe clinical progression within 3 days of COVID-19 clinical onset to receive sulodexide 1000 lipase releasing units/day or placebo for 21 days. Sulodexide is a natural glycosaminoglycan composed of 80% fast moving heparin plus 20% dermatan sulfate [60]. Ιts in vitro antihemostatic effects have been shown to be at least comparable with those of enoxaparin [61]. The authors concluded that patients treated with sulodexide had a significantly lower risk for hospitalization and supplemental oxygen need along with improved laboratory parameters without significantly increased major bleeding risk. The ACTIV-4B COVID-19 Outpatient Thrombosis Prevention Trial studied symptomatic but clinically stable outpatients receiving aspirin or therapeutic or prophylactic dose of apixaban or no anticoagulation [52]. The trial was terminated early due to low event rates and failed to conclude if there are improvements in clinical outcomes in the aspirin or apixaban groups over no anticoagulation in outpatients. The OVID study randomized 472 outpatients to receive prophylactic enoxaparin dosage versus standard of care (no thromboprophylaxis) and showed a similar risk of hospitalization and death between the two treatment arms. Similar to the ACTIV-4B study, the OVID study was terminated early due to low event rates and failed to conclusively assess the futility of thromboprophylaxis under the initial study design assumptions. The same results and conclusions were reached from the investigators of the ETHIC study that randomized 219 outpatients to a prophylactic dose of enoxaparin versus standard of care (no thromboprophylaxis) [58]. The ETHIC study was also terminated early due to low event rates.

The Medically Ill Hospitalized Patients for COVID-19 Thrombosis Extended ProphyLaxis With Rivaroxaban Therapy (MICHELLE) trial randomized post-discharge patients at increased risk for VTE (International Medical Prevention Registry on Venous Thromboembolism [IMPROVE] VTE score of ≥4 or 2–3 with a D-dimer >500 ng/mL) to rivaroxaban 10 mg/day or no anticoagulation for 35 days [56]. Results demonstrated a reduction in the composite endpoint of major thromboembolic events and cardiovascular mortality in the prophylactic group and overall no major bleeding risk in either group. The authors concluded in favor of the use of prophylactic dosages of rivaroxaban in high-risk post-discharge patients.
***Conclusion-Recommendation:******Outpatients:****Available data indicate against routine pharmacologic thromboprophylaxis in outpatients with COVID-19 in general.**It is reasonable to suggest individualized thromboprophylaxis in outpatients at high risk for disease worsening (with adverse prognostic factors for severe disease, potential candidates for hospitalization or “hospital-at home programs”) and/or increased VTE risk after careful assessment of the bleeding risk.**Regular assessment and reevaluation for disease worsening and bleeding risk is strongly recommended.****Post-discharge:****Post-hospital discharge prophylactic anticoagulation with rivaroxaban 10 mg once daily for approximately 1 month is recommended in high VTE risk patients if no drug-drug interactions are expected.*

**Table 1:** Randomized Controlled Trials regarding thromboprophylaxis strategies in patients with COVID-19.

Hospitalized						
Study	N	Setting	Comparator	Intervention	Findings	
					Efficacy	Safety
X-COVID-19 [44]	183	General Wards	Prophylactic Enoxaparin	Intermediate Enoxaparin	Intermediate: ↓ PE No DVT in both groups (underpowered study/premature discontinuation)	↔ Major bleedings
HEP-COVID [37]	253	D-dimer > 4 ULN or SIC score ≥ 4 ICU 33%	Prophylactic or Intermediate LMWH/UFH	Therapeutic Enoxaparin	Therapeutic: ↓ VTE/ATE/Death	↔ Major bleedings
RAPID [38]	465	General Wards (moderately ill + increased D-dimer)	Prophylactic LMWH/UFH	Therapeutic LMWH/UFH	Therapeutic: ↓ Death	↔ Major bleedings
Perepu et al. [43]	176	ICU and/or coagulopathy ^†^ ICU 62%	Prophylactic Enoxaparin	Intermediate Enoxaparin	↔ VTE/ATE/Death	↔ Major bleedings
ACTION [24]	615	Hospitalized + increased D-dimer ICU 6%	Prophylactic Enoxaparin/UFH (mainly Enoxaparin)	Extended Therapeutic Rivaroxaban/Enoxaparin/UFH (mainly Rivaroxaban)	↔ Duration of hospitalization or oxygen supply/VTE/ATE/Death	Therapeutic: ↑ Bleeding events
INSPIRATION [32]	562	ICU 100%	Prophylactic Enoxaparin	Intermediate Enoxaparin	↔ VTE/ATE/ECMO/Death	↔ Major bleeding
HESACOVID [39]	20	IMV ICU 100%	Prophylactic Enoxaparin/UFH	Therapeutic Enoxaparin	Therapeutic: ↑ PaO_2_/FiO_2_ ↓ need for IMV	↔ Major bleeding
Oliynyk et al. [40]	126	Severely ill ICU 100%	Prophylactic Enoxaparin	Therapeutic Enoxaparin/UFH	Therapeutic enoxaparin/UFH: ↓ intubation/death	↔ Major bleeding
REMAP-CAP, ACTIV-4a and ATTACC Critically ill [36]	1098	Critically ill ICU 100%	Prophylactic or Intermediate LMWH/UFH	Therapeutic LMWH/UFH	↔ VTE/ATE/Organ support-free days/Death (premature discontinuation—futility)	↔ Major bleedings
REMAP-CAP, ACTIV-4a and ATTACC Non-critically ill [35]	2219	Non-critically ill ICU 0%	Prophylactic or Intermediate LMWH/UFH	Therapeutic LMWH/UFH	Therapeutic: ↑ Organ support–free days ↓ Death (premature discontinuation—superiority)	↔ Major bleeding
COVID-HEP [41]	159	Acutely ill + increased D-dimer or critically ill ICU 28%	Prophylactic (acutely) or Intermediate (critically) enoxaparin/UFH	Therapeutic enoxaparin/UFH	↔ VTE/ATE/DIC/Death(premature discontinuation—low recruitment rate)	↔ Major bleeding
BEMICOP [42]	65	General Wards (moderately ill + increased D-dimer)	Prophylactic Bemiparin	Therapeutic Bemiparin	↔ VTE/ATE/development of ARDS/Need for mechanical ventilation support/ICU admission/Death	↔ Major bleeding
RECOVERY [49]	14892	Hospitalized * ICU 5%	Standard of Care	Standard of care + Aspirin 150 mg	↔ Progressing to IMV or Death	Aspirin: ↑ Major bleeding
REMAP-CAP [50]	1557	Critically ill ^§^ ICU 100%	No antiplatelet therapy	Aspirin or P2Y12 inhibitor	↔ Organ support-free days (premature discontinuation—futility)	Antiplatelets: ↑ Major bleeding
ACTIV-4a [51]	562	Non critically ill ICU 0%	Therapeutic Heparin	Therapeutic Heparin + P2Y12 inhibitor	↔ VTE/ATE//Organ support-free days/Death	↔ Major bleeding
**Non-Hospitalized**						
**Study**	**N**	**Setting**	**Comparator**	**Intervention**	**Findings**	
					**Efficacy**	**Safety**
Gonzalez-Ochoa et al. [57]	243	Outpatients at high risk for severe clinical progression within 3 days of COVID-19 clinical onset	Placebo	Sulodexide (oral 1000 LRU/d) for 21 days	Sulodexide: ↓ Hospitalization/supplementary oxygen need/d-dimer/CRP	↔ Major bleeding
ACTIV-4B [52]	657	Symptomatic but clinically stable outpatients	Placebo	Prophylactic Apixaban (2.5 mg twice daily) Therapeutic Apixaban (5 mg twice daily) Aspirin (81 mg once daily)	↔ VTE/ATE/Hospitalization/Death (premature discontinuation—low event rate)	↔ Major bleeding
ETHIC [58]	219	Outpatients ≥ 30 years with symptomatic COVID-19 + one risk factor for severe disease	Standard of Care (No thromboprophylaxis)	Prophylactic Enoxaparin	↔ Hospitalization/Death (premature discontinuation—low event rate)	↔ Major bleeding
OVID [59]	472	Outpatients ≥ 50 years with respiratory symptoms and body temperature > 37.5 °C	Standard of Care (No thromboprophylaxis)	Prophylactic Enoxaparin	↔ Hospitalization/Death (premature discontinuation—low event rate)	↔ Major bleeding
MICHELLE [56]	320	Post-discharge with increased VTE risk ^¶^	Prophylactic Rivaroxaban (10 mg) for 35 days	No anticoagulation	Rivaroxaban: ↓ VTE/ATE/Death	↔ Major bleeding

ARDS, acute respiratory distress syndrome; ATE, arterial thromboembolism; CRP, C-reactive protein; DIC, disseminated intravascular coagulopathy, DVT; Deep vein thrombosis; ECMO, extracorporeal membrane oxygenation; ICU, intensive care unit; IMV, invasive mechanical ventilation; P2Y12 inhibitor, clopidogrel, ticagrelor or prasugrel; LMWH; Low molecular weight heparin; LRU, lipase releasing units; PE; Pulmonary embolism; SIC, sepsis-induced coagulopathy; UFH, Unfractionated heparin; ULN, upper limit of normal; VTE, venous thromboembolism; * Patients were already receiving anticoagulation thromboprophylaxis (33% high LMWH dosage, 60% prophylactic LMWH, 7% no anticoagulation); ^†^ Coagulopathy defined as modified (International Society on Thrombosis and Haemostasis) ISTH Overt disseminated intravascular coagulation (DIC) score ≥ 3; ^§^ Patients were already receiving anticoagulation thromboprophylaxis (therapeutic 11%, intermediate 58%, prophylactic 18%, unknown 13%); ^¶^ All patients received prophylactic LMWH/UFH/fondaparinux during hospitalization. ↑, Intervention increased the endpoint versus comparator; ↓, Intervention decreased the endpoint versus comparator; ↔, No difference in the endpoint between intervention and comparator.

**Table 2:** Randomized Clinical Trials evaluating the optimal dosage of thromboprophylaxis in hospitalized patients with COVID-19.

Study	N	ICU (%)	Comparator	Intervention	Result-Conclusion
X-COVID-19 [44]	183	0	Prophylactic Enoxaparin	Intermediate Enoxaparin	Underpowered Fewer pulmonary embolism events with Intermediate
HEP-COVID [37]	253	33	Prophylactic or Intermediate LMWH/UFH	Therapeutic Enoxaparin	Improved clinical outcomes with Therapeutic only in non-ICU patients
RAPID [38]	465	0	Prophylactic LMWH/UFH	Therapeutic LMWH/UFH	Fewer deaths with Therapeutic
Perepu et al. [43]	176	62	Prophylactic Enoxaparin	Intermediate Enoxaparin	No difference
ACTION [24]	615	6	Prophylactic Enoxaparin/UFH (mainly Enoxaparin)	Extended Therapeutic Rivaroxaban /Enoxaparin/UFH (mainly Rivaroxaban)	No difference
INSPIRATION [32]	562	100	Prophylactic Enoxaparin	Intermediate Enoxaparin	No difference
HESACOVID [39]	20	100	Prophylactic Enoxaparin/UFH	Therapeutic Enoxaparin	Improved oxygenation parameters with Therapeutic
Oliynyk et al. [40]	126	100	Prophylactic Enoxaparin	Therapeutic Enoxaparin/UFH	Improved clinical outcomes with Therapeutic
REMAP-CAP, ACTIV-4a and ATTACC Critically ill [36]	1098	100	Prophylactic or Intermediate LMWH/UFH	Therapeutic LMWH/UFH	No difference
REMAP-CAP, ACTIV-4a and ATTACC Non-critically ill [35]	2219	0	Prophylactic or Intermediate LMWH/UFH	Therapeutic LMWH/UFH	Improved clinical outcomes with Therapeutic
COVID-HEP [41]	159	28	Prophylactic (acutely) or Intermediate (critically) enoxaparin/UFH	Therapeutic enoxaparin/UFH	No difference
BEMICOP [42]	65	0	Prophylactic Bemiparin	Therapeutic Bemiparin	No difference

ICU, intensive care unit; LMWH; Low molecular weight heparin; UFH, Unfractionated heparin.

## 4. Conclusions

Thromboprophylaxis has been regarded as one of the most important therapeutic interventions for patients with COVID-19 since the onset of the pandemic. Most guidance recommendations have been primarily based on data derived from observational studies. Recently, high quality RCTs have been published shedding light on the optimal strategies that should be followed. Careful interpretation and implementation of their findings should be the cornerstone of the physicians’ practices in addressing everyday clinical problems and providing the best health services to these patients. LMWH represents the most well-studied type of thromboprophylaxis in hospitalized patients. At present, current randomized data support the use of therapeutic dosage in acutely ill non-ICU or ward patients with high thrombotic risk, discourage escalation strategy with worsening status, and suggest prophylactic dosage in critically ill patients, especially in the ICU. Yet, the role of the intermediate dosage in high thrombotic risk hospitalized patients without critical disease (non-ICU) has not been extensively studied in the context of RCTs. Thromboprophylaxis should not be routinely administered in outpatients; however thromboprophylaxis should be administered on an individualized basis in post-discharge patients with COVID-19 with high thrombotic risk. Moreover, the change in the dominant SARS-CoV-2 variants, the wide immunization status (increasing rates of vaccination and natural immunity), and the availability of antiviral therapies and monoclonal antibodies in the outpatient setting might affect the characteristics of the patients with COVID-19; thus, further studies are needed for the optimal management of their thrombotic risk.

## Data Availability

Not applicable.

## Supplementary Materials

The following supporting information can be downloaded at: https://www.mdpi.com/article/10.3390/jcm11205997/s1, Members of COVID-19 Thrombosis Collaborative Group.
