# Supplementary material for: Practical Recommendations for Optimal Thromboprophylaxis in Patients with COVID-19: A Consensus Statement Based on Available Clinical Trials
Source: J Clin Med. 2022 Oct 11;11(20):5997. doi: 10.3390/jcm11205997 (PMC9604499; doi:10.3390/jcm11205997)
Supplement: Supplementary file 1 [file jcm-11-05997-s001.zip › jcm-1926193-supplementary.pdf]

COVID-19 Thrombosis Collaborative Group

No	Name	Affiliation	E-mail	COI
1	Darko Antic	• Clinic for Hematology, University Clinical Center Serbia, Belgrade, Serbia<br>• Faculty of Medicine, University of Belgrade, Belgrade, Serbia	darko.antic1510976@gmail.com	-
2	Aleš Blinc	Department of Vascular Diseases, Division of Internal Medicine, University Medical Centre, Ljubljana, Slovenia	ales.blinc@kclj.si	-
3	Francesco Boccardo	Department of Surgical Sciences (DISC), Unit of Surgical Lymphology, University of Genoa, Italy	francesco.boccardo@unige.it	-
4	Marianne Brodmann	Division of Angiology, Department of Internal Medicine, Medical University, Graz, Austria	marianne.brodmann@medunigraz.at	-
5	Varusca Brusegan	Department of Immunohematology and Transfusion Medicine, Hospital Papa Giovanni XXIII, Bergamo, Italy.	vbrusegan@asst-pg23.it	-
6	Alexandra Canedo	Serviço de Angiologia e Cirurgia Vascular, Centro Hospitalar de Vila Nova de Gaia e Espinho, Gaia, Portugal	canedoalexandra@gmail.com	-
7	Denisa Čelovská	1st Dpt. of Internal Medicine, Faculty of Medicine, Comenius University, Bratislava, Slovakia	denisa.celovska@gmail.com	-
8	Benilde Cosmi	Division of Angiology and Blood Coagulation, Department of Specialty, Diagnostics and Experimental Medicine, S.Orsola-Malpighi University Hospital Research Institute IRCCS<br>University of Bologna, Bologna Italy	benilde.cosmi@unibo.it	Advisory board of VIATRIS, TECHDOW PHARMA ITALY, received speaker fees for Daiichi Sankyo, Sanofi, Instrumentation Laboratory.
9	Sergio De Marchi	Division of Angiology, Department of Medicine, University of Verona, Verona, Italy	sergio.demarchi@univr.it	-
10	Gabriel Dimitrov	Department of Cardiovascular Medicine, Cardiology-Angiology Division, L. Sacco Hospital, Milan, Italy	gdimitrov@libero.it	-
11	Katalin Farkas	Department of Angiology, Szent Imre University Teaching Hospital, Budapest, Hungary	farkask@hotmail.com	-
12	Anders Gottsäter	Department of Medicine, Lund University, Malmö, Sweden	anders.gottsater@med.lu.se	-
13	Christian Heiss	• Department of Clinical and Experimental Medicine, University of Surrey, Guildford, UK<br>• Vascular Department, Surrey and Sussex Healthcare NHS Trust, Redhill, UK	c.heiss@surrey.ac.uk	-
14	Oguz Karahan	Medicalpark School Alanya Alaaddin Keykubat University, Department of Cardiovascular Surgery,Alanya, Turkey	oguzk2002@gmail.com	-
15	Endre Kolossváry	Department of Angiology, St. Imre University Teaching Hospital, Budapest, Hungary	kolossendre@gmail.com	-

**16**	Matija Kozak	Department of Vascular Diseases, Division of Internal Medicine, University Medical Centre, Ljubljana, Slovenia	matijakozak@gmail.com	-
**17**	Abraham A Kroon	Department of Internal Medicine, Section Vascular Medicine, Maastricht University Medical Center, Cardiovascular Research Institute Maastricht (CARIM), Maastricht University, Maastricht, The Netherlands	aa.kroon@mumc.nl	-
**18**	Gianfranco Lessiani	Angiology Unit, Department of Internal Medicine, Città S'Angelo Hospital (PE) Italy	gf.lessiani@gmail.com	-
**19**	Aaron Liew	• Portiuncula University Hospital, Saolta University Health Care Group, Galway, Ireland<br>• School of Medicine, National University of Ireland Galway (NUIG), Galway, Ireland	aaron.liew@nuigalway.ie	-
**20**	George Marakomichelakis	Fourth Department of Internal Medicine and Unit for Medical Angiology, Evangelismos State General Hospital, Athens, Greece	gmarakom@gmail.com	-
**21**	Peter Marschang	Department of Internal Medicine, Central Hospital of Bolzano (SABES-ASDAA), Bolzano, Italy	peter.marschang@sabes.it	-
**22**	Lucia Mazzolai	Heart and Vessel Department, Division of Angiology, Lausanne University Hospital (CHUV), Lausanne, Switzerland	lucia.mazzolai@chuv.ch	-
**23**	Nikolaos-Georgios Papageorgiou	National and Kapodistrian University of Athens, School of Medicine, Third Department of Medicine, Sotiria Hospital, Athens, Greece	ngpapageorgiou@gmail.com	-
**24**	Sergio Pillon	UOSD Angiology, San Camillo-Forlanini Hospital, National Health Institute ISS, AO San Camillo Forlanini, Rome, Italy	pillon@gmail.com	-
**25**	Pavel Poredos	University Clinical Center Ljubljana, Slovenija	pavel.poredos@kclj.si	-
**26**	Dusan Suput	Faculty of Medicine - University of Ljubljana, Institute of Pathophysiology, Slovenia	dusan.suput@mf.uni-lj.si	-
**27**	Marc Righini	Division of Angiology and Haemostasis, Department of Medicine, Geneva University Hospitals, Geneva, Switzerland	marc.righini@hcuge.ch	-
**28**	Gerit-Holger Schernthaner	• Division of Angiology, Department of Medicine II, Medical University & General Hospital Vienna, Vienna, Austria<br>• European Academy of Sciences and Arts, Salzburg, Austria<br>• VAS, European Independent Foundation, Milano, Italy	gerit.schernthaner@meduniwien.ac.at	-
**29**	Aleksander Sieroń	Faculty of Health Sciences, Jan Dlugosz University, Czestochowa, Poland	asieron2@wp.pl	-
**30**	Jonas Spaak	Department of Clinical Sciences, Danderyd University Hospital, Karolinska Institutet, Stockholm, Sweden.	jonas.spaak@ki.se	Received speaker honoraria from NovoNordisk, Novartis, Bayer and AstraZeneca
**31**	Muriel Sprynger	Department of Cardiology, University of Liège Hospital, Liege, Belgium	msprynger@chuliege.be	Advisory board of BMS-PFIZER,

				BAYER and DAIICHY-SANKYO
**32**	Agata Stanek	Department and Clinic of Internal Medicine, Angiology and Physical Medicine, Faculty of Medical Sciences in Zabrze, Medical University of Silesia, Zabrze, Poland	astanek@tlen.pl	Conducted lectures for Bayer and alfassigma
**33**	Daniel Staub	Angiology/Vascular Medicine, Medical Head Department of Circulation, Thorax and Transplantation, University Hospital Basel, Basel, Switzerland	daniel.staub@usb.ch	-
**34**	Igor Stojkovski	• Faculty of Medicine, Ss. Cyril and Methodius University, Skopje, North Macedonia<br>• University Clinic of Radiotherapy and Oncology, Skopje, North Macedonia	igor.stojkovski@medf.ukim.edu.mk	-
**35**	Adriana Visonà	Angiology Unit, Department of Vascular Medicine, Castelfranco Veneto (Treviso), Italy	adrianavisona@gmail.com	-
**36**	Dragan Vasic	Department of Noninvasive Vascular Laboratory, Clinic of Vascular and Endovascular Surgery, University Clinical Centre of Serbia, Belgrade, Serbia	dr_dragan_vasic@yahoo.com	-
**37**	Jean-Claude Wautrecht	Department of Vascular Diseases, Hôpital Erasme, Université Libre de Bruxelles, Brussels, Belgium	jean.claude.wautrecht@erasme.ulb.ac.be	-
**38**	Andrea Willfort-Ehringer	Division of Internal Medicine, Department of Angiology, Medical University of Vienna, Vienna, Austria	andrea.willfort@gmail.com	-
**39**	Isabelle Durand-Zaleski	Université de Paris, CRESS, INSERM, INRA, URCEco, AP-HP, Hôpital de l'Hôtel Dieu, Paris, France	isabelle.durand-zaleski@aphp.fr	-
**40**	Pecsvarady Zsolt	Second Department of Internal Medicine (Vascular Center), Flor Ferenc Teaching Hospital, Budapest, Hungary	pecsvarady@gmail.com	-
